# A transcriptomic snapshot of early molecular communication between *Pasteuria penetrans* and *Meloidogyne incognita*

**DOI:** 10.1186/s12864-018-5230-8

**Published:** 2018-11-29

**Authors:** Victor Phani, Vishal S. Somvanshi, Rohit N. Shukla, Keith G. Davies, Uma Rao

**Affiliations:** 1 Division of Nematology, ICAR-Indian Agricultural Research Institute, New Delhi, India; 2 Bionivid Technology Private Limited, 209, 4th Cross, Kasturi Nagar, Bangalore, India; 3 Department of Biological and Environmental Sciences, University of Hertfordshire, Hatfield, UK; 4 Division of Biotechnology and Plant Health, Norwegian Institute of Bioeconomy Research, Postboks 115 NO-1431 Ås, Norway

**Keywords:** Endospores, *Meloidogyne incognita*, *Pasteuria penetrans*, Root-knot nematode, Transcriptome

## Abstract

**Background:**

Southern root-knot nematode *Meloidogyne incognita* (Kofoid and White, 1919), Chitwood, 1949 is a key pest of agricultural crops. *Pasteuria penetrans* is a hyperparasitic bacterium capable of suppressing the nematode reproduction, and represents a typical coevolved pathogen-hyperparasite system. Attachment of *Pasteuria* endospores to the cuticle of second-stage nematode juveniles is the first and pivotal step in the bacterial infection. RNA-Seq was used to understand the early transcriptional response of the root-knot nematode at 8 h post *Pasteuria* endospore attachment.

**Results:**

A total of 52,485 transcripts were assembled from the high quality (HQ) reads, out of which 582 transcripts were found differentially expressed in the *Pasteuria* endospore encumbered J2 s, of which 229 were up-regulated and 353 were down-regulated. *Pasteuria* infection caused a suppression of the protein synthesis machinery of the nematode. Several of the differentially expressed transcripts were putatively involved in nematode innate immunity, signaling, stress responses, endospore attachment process and post-attachment behavioral modification of the juveniles. The expression profiles of fifteen selected transcripts were validated to be true by the qRT PCR. RNAi based silencing of transcripts coding for fructose bisphosphate aldolase and glucosyl transferase caused a reduction in endospore attachment as compared to the controls, whereas, silencing of aspartic protease and ubiquitin coding transcripts resulted in higher incidence of endospore attachment on the nematode cuticle.

**Conclusions:**

Here we provide evidence of an early transcriptional response by the nematode upon infection by *Pasteuria* prior to root invasion. We found that adhesion of *Pasteuria* endospores to the cuticle induced a down-regulated protein response in the nematode. In addition, we show that fructose bisphosphate aldolase, glucosyl transferase, aspartic protease and ubiquitin coding transcripts are involved in modulating the endospore attachment on the nematode cuticle. Our results add new and significant information to the existing knowledge on early molecular interaction between *M. incognita* and *P. penetrans*.

**Electronic supplementary material:**

The online version of this article (10.1186/s12864-018-5230-8) contains supplementary material, which is available to authorized users.

## Background

Following the publication of the *Caenorhabditis elegans* (Maupas, 1900) Dougherty, 1955 genome [104], some laboratories have become focused on using this nematode as a model for studying innate immunity [61, 71, 96, 111]. Due to the short developmental time of this nematode, the studies have necessarily focused on the infection of adults and earlier developmental stages, and in particular the non-feeding dauer stage have been neglected. The life-cycle of plant-parasitic nematodes commences when an infective juvenile hatches from an egg as a second-stage juvenile, and migrate through the soil seeking a suitable host plant before feeding starts. This period of time offers the opportunity to study early responses of the nematode to bacterial infection [26].

*Pasteuria penetrans* (Thorne, 1940) Sayre and Starr, 1985, a Gram-positive soil bacterium of the *Bacillus*–*Clostridium* clade, is a hyperparasite of the root-knot nematodes (RKN), and represents a typical naturally coevolved pathogen – hyperparasite system [15, 86]. This is an excellent system to study the early stages of the nematode infection processes by bacterial parasites. The life-cycle and developmental stages of *P. penetrans* inside *M. incognita* are well documented and understood [28, 82]. The bacterium completes its life-cycle in three phases, [1] Attachment and germination, [2] Rhizoid production and exponential growth; and [3] Sporogenesis [28]. There is a high degree of genetically regulated host specificity involved in this interaction. *Pasteuria* is highly selective and specific to their host; one population of *Pasteuria* will not recognize and infect other species in the same genus, and not even all populations of the same species [24]. The surface of nematode cuticle plays a decisive role in facilitating the specificity of the adhesion [27, 99] and the attachment of *P. penetrans* endospores to an as of yet uncharacterized cuticle receptor is the primary and arguably the most crucial step of the bacterial infection [28]. After the RKN J2 s establish permanent feeding sites in their plant hosts, the endospores perceive some currently unknown cue(s) from the nematode and germinate [25, 93]. The bacterium proliferates inside the worm’s body, kills it, and converts the females into an “endospore sac” containing millions of endospores [25, 82].

The recent development of genomic tools and technologies for the plant-parasitic nematodes has enabled researchers to investigate in detail at the molecular level the nematode’s interactions with their hosts, symbionts and pathogens/hyperparasites. It is known that hosts respond to pathogen attack by altering their gene expression; in the infection of *Daphnia* by *Pasteuria ramosa*, it was observed that the incompatible/resistant hosts responded by an up-regulated gene response, whereas the down-regulated gene response was pronounced in the compatible/susceptible host [64]. However, in a recently published study that used RNA-Seq to understand the nematode’s response to *Pasteuria* infection at three days post attachment, when nematode appeared less mobile and moribund because of the bacterial infection, it was found that 91% of the 445 differentially expressed genes were up-regulated [117]. This was contrary to the general understanding that a down-regulated gene response is exhibited by the susceptible hosts, which is probably caused by subversion of host immunity by the parasite [6]. Therefore, this large up-regulation in gene expression by *Daphnia* against *Pasteuria* infection over time, as reported by McTaggart et al. [64] and again by Zou et al. [117] using a population of *M. incognita* susceptible to *Pasteuria penetrans* at three days post attachment warranted further investigation.

In order to understand the nematode genes involved in the very first and crucial stage of the *M. incognita* – *P. penetrans* interaction, i.e. the recognition and adhesion of endospores, here we investigated the early transcriptional response of *M. incognita* at eight hours after their initial encounter and before the infective J2 s have invaded the plant root. Additionally, we also identified the functional role of five important differentially expressed genes in *M. incognita* – *Pasteuria* interaction by using RNAi based gene silencing and measuring their effects on endospore adhesion.

## Methods

### Biological materials

The single egg mass culture of an Indian isolate of *M. incognita* race 1 was increased on tomato plant (*Solanum lycopersicum* L. cv. Pusa ruby) in a glasshouse at ICAR- Indian Agricultural Research Institute, New Delhi, India. Nematode infected tomato roots were washed free of soil, egg mass were hand-picked and kept for hatching on a modified Baermann’s funnel assembly [110]. The freshly hatched J2 s were used for all of the experiments, viz., endospore attachment, RNA isolation, dsRNA treatment etc. The unused/left over J2 s were autoclaved and discarded.

### Endospore attachment

*Pasteuria penetrans* (Strain AII-329: *Pasteuria* collection, ICAR-IARI, New Delhi, India) endospores were produced on *M. incognita* cultured on adzuki bean (*Vigna angularis* (Willd.) Ohwi and Ohashi) in CYG growth pouches (Mega International, St Paul, MN, USA) as described by Rao et al. [88]. Transfer of germinated seeds in growth pouches, setting up of root infection and post infection maintenance of plants were conducted as described earlier [84].

The freshly hatched ca. 20,000 J2 s were collected in a 1.5 ml microcentrifuge tube and mixed with 200 μl of endospore suspension (2.5 × 10^3^ endospores ml^− 1^). The attachment of endospores was pursued by centrifugation method [41] and resulted in attachment of approximately 30–35 endospores on the cuticle surface of each juvenile (*n* = 200). The attachment of endospores onto the juveniles was confirmed microscopically. Following endospore attachment, the J2 s were washed thrice in M9 buffer (1 mM MgSO_4_, 22 mM KH_2_PO_4_, 42.3 mM Na_2_HPO_4_ and 85.6 mM NaCl; pH 7.0) to remove the free endospores. The endospore encumbered J2 s were incubated in fresh M9 buffer at room temperature (28 °C) for 8 h on a slowly moving rotator. The adherence of the endospores onto J2 surface was again confirmed microscopically after 8 h, and the juveniles were found to have similar numbers of attached spores as seen earlier. Following attachment, the J2 s were incubated at room temperature (28 °C) for 8 h on a slowly rotating incubator. The freshly hatched juveniles incubated in M9 buffer for 8 h without *Pasteuria* endospores served as a control.

### RNA extraction

Total RNA was extracted from about 20,000 *M. incognita* non-encumbered and endospore encumbered J2 s with TRIzol reagent (Thermo Fisher Scientific, Waltham, MA, USA) according to manufacturer’s protocol. The RNA was treated with RQ1 RNase-Free DNase (Promega, Madison, WI, USA) to remove any genomic DNA contamination. The integrity of the isolated RNA was tested on a Bioanalyzer (Agilent Technologies, Santa Clara, CA, USA). The quality and concentration was determined by 1% agarose gel and NanoDrop-1000 spectrophotometer (Thermo Fisher Scientific, Waltham, MA, USA). The process was replicated twice.

### cDNA synthesis, library preparation, RNA-sequencing

The total RNA was subjected to downstream processing for cDNA synthesis and library preparation. The extracted RNA was assessed for quality using an Agilent 2100 bioanalyzer (Agilent Technologies, Santa Clara, CA, USA) and RNA with an RNA integrity number (RIN) of 8.0 was used for mRNA purification. The mRNA (messenger RNA) was purified from approximately 5 μg of intact total RNA using oligodT beads (Illumina® TruSeq® RNA Sample Preparation Kit v2). The purified mRNA was fragmented in the presence of bivalent cations and first strand cDNA was synthesized using Superscript II reverse transcriptase (Invitrogen, Carlsbad, CA, USA) and random hexamer primers (Invitrogen, Carlsbad, CA, USA). Second strand cDNA was synthesized in the presence of DNA polymerase I and RNaseH following standard protocol (Illumina). The cDNA was cleaned using Agencourt AMPure XP purification kit (Beckman-Coulter, Brea, CA, USA), amplified, quantified using a Nanodrop spectrophotometer (Thermo Fisher Scientific, Waltham, MA, USA) and checked for quality with a Bioanalyzer (Agilent Technologies, Santa Clara, CA, USA). In total, 4 libraries were prepared for non-encumbered and *Pasteuria* encumbered samples (2 each) as per the Illumina protocols. The cDNA libraries were then sequenced on the Illumina HiSeq platform by outsourcing to Bionivid Technologies Pvt. Ltd., Bangalore, India.

### Transcriptome assembly, quantitation and identification of differentially expressed transcripts

All the Paired End fastq files were subjected to standard quality control using NGS QC toolkit, v2.3.3 [77]. Reads with adapter contamination were removed along with their mate pair. High Quality (HQ) reads from all the samples were merged together to generate a Primary Assembly using Trinity Assembler (Trinity RNA-Seq-v2.0.6) [36] with default *k-mer* length 25, minimum contig length 200 bp and minimum *k-mer* coverage as 5. Further amelioration of the transcripts was done by filtering on the basis of average depth (≥ 5) and coverage (≥ 70%) in the individual samples [5]. The ameliorated transcripts were then subjected to clustering using CD_HIT_EST (v4.6.1) to make the secondary and final assembly with sequence identity threshold as 0.8 and length difference cut off as 0.9. The redundant transcripts were removed by CD_HIT_EST to make the secondary assembly. We observed a higher percentage (~ 45%) of small transcripts (< 500 bp). These were further fileterd based on annotation obtained against NCBI NRDB protein database. Sequences < 500 bp, which remained un-annotated, were discarded and a final transcriptome assembly was generated.

The final assembly was used for quantitation of transcripts in each of the individual libraries by using RSEM method [58]. The assembly validated .bam (Binary Sequence Alignment/Map) file was processed using bedtools [87] and samtools [59] for quantitation (read count estimation) for each transcript in a library and also to calculate the total coverage and average depth of the transcriptome in each library. The differentially expressed transcripts were identified using DESeq R package [3] between the treatment (endospore encumbered J2 s) and control (non-encumbered J2 s) groups in replicate. The differential expression of transcripts was determined with log2fold change ≥2 & *P* value ≤0.05 obtained by DESeq analysis.

### Transcript annotation

Homology based annotation for the final transcriptome was done against National Center for Biotechnology Information (NCBI) non-redundant (nr) protein database [2]. The filtration criteria used for blastx were: Evalue ≤0.001, Query Coverage ≥60 and Percentage Identity ≥40. The results were subjected to Gene Ontology (GO) and Pathway analysis using Blast2GO [22] and KAAS [69]. Additional analysis were performed to find the secreted peptides, neuropeptides and RNAi pathway genes present in the differentially expressed transcripts by using SignalP v4.1 [72], and by blast search against the local database of *C. elegans* neuropeptide sequences (Li and Kim, 2008) and *M. incognita* RNAi genes [1] at E value ≤0.001 and query coverage ≥60, respectively.

### Validation of RNA-Seq gene expression data by qRT PCR

Quantitative real time PCR (qRT PCR) was carried out to confirm the expression pattern of fifteen selected transcripts differentially expressing in the range of + 2.01 fold to − 5.06 fold in the RNA-Seq experiment. These 15 transcripts (Table 1) were chosen based on their predicted role in nematode – *Pasteuria* interaction, and included ten down-regulated transcripts and five up-regulated transcripts. cDNA was prepared from the same RNA samples that were used for RNA-Seq. Approximately 500 ng of RNA was reverse transcribed using cDNA synthesis kit (Superscript VILO, Invitrogen, Carlsbad, CA, USA) and qRT PCR was performed in a realplex^2^ thermal cycler (Eppendorf, Hamburg, Germany) using SYBR Green Supermix Kit (Eurogentec, Liege, Belgium). Each reaction mixture contained a final volume of 10 μl, comprised of 5 μl of SYBR Green PCR Master mix (Eurogentec, Liege, Belgium), 750 nM of each primer and 1.5 ng of cDNA. To normalize the gene expression level 18S rRNA (Genbank accession: HE667742), a constitutively expressed gene was used as internal reference. Three biological and three technical replicates were maintained for each sample. The data were analyzed by ΔΔCt method [60] and results were expressed as log2-transformed fold change values and Dunnet’s multiple comparison was performed for determining the statistical significance of the expression data. The primer details for qRT PCR are provided in Table 1.

**Table 1:** List of primers used in this study

S. No.	Transcript name	Primer names	Annotation	Primer sequence (5′ - 3′)	Product length (bp)	Tm (°C)	Purpose
1	TR11426	P1_F	heat shock protein 20	GGAAGAGGAACACAATGGCA	112	60	qRT PCR
		P1_R		TGCCTCAAATTTCCCAGTCC			
2	TR14120	P3_F	phospholipase A2	ACATTTCTCCATGTCAGCAC	78	60	qRT PCR
		P3_R		CGTTGCACTGGAGGAATAAA			
3	TR16177	P4_F	aspartic protease	CTCCCTATCCTCCACCTATCAA	104	60	qRT PCR
		P4_R		CACCAAAGCTGACGGTATCA			
4	TR10194	P5_F	Bm 3887	AAACCTGGCAGATCACAAC	106	60	qRT PCR
		P5_R		CTCTGCTGTACCACAAACAA			
5	TR10010	P6_F	fructose bis phosphate aldolase	GACCACCAGATAGGAATACAAC	114	60	qRT PCR
		P6_R		GGCAATCTTCACCCAAGAA			
6	TR31579	P7_F	selenium binding protein	ATATATGAAGGTGGCCCTTGTC	131	60	qRT PCR
		P7_R		GCAATTGAAGAACCGACTTCTG			
7	TR14793	P8_F	glucosyl transferase	CCATTTGACCACTCGATTCA	107	60	qRT PCR
		P8_R		GCATATCGCTCCTCAAATCA			
8	TR23171	P10_F	venom Allergen like protein	TTGGACGTTGCCCTAGATA	100	60	qRT PCR
		P10_R		CTACATGGCTCACCAACATT			
9	TR35213	P11_F	glycoside hydrolase	GGTGATTCCACCAGCATATT	121	60	qRT PCR
		P11_R		CCAAATGGCCCAGTATCTT			
10	TR40461	P12_F	glutathione S transferase	TAAGCCAGAAGAGCCGAAA	111	60	qRT PCR
		P12_R		GTGGATCAACTTCGAAAGACTG			
11	TR11544	P13_F	fatty acid and retinol binding protein	CGAATTGACCGAAGATGACA	106	60	qRT PCR
		P13_R		TTCGCTCTTCTCCTTCAATG			
12	TR26363	P14_F	major sperm protein	ATACGTCGCGGTCTACAA	134	60	qRT PCR
		P14_R		TTCCGCTTCCGTCCTATT			
13	TR10990	P15_F	ubiquitin	CCTCGACTGTTCGTGTATTG	101	60	qRT PCR
		P15_R		GTCATCATCCAACTGACATCC			
14	TR20164	P17_F	tropomyosin	CGGGCAACCTCATCATATT	108	60	qRT PCR
		P17_R		GAACCGCTCGTTACAAGAT			
15	TR24005	P18_F	serine protease	GGGTCATTCGTGCCATTT	108	60	qRT PCR
		P18_R		TGGTAATACGACCGTCTACTC			
16	18S rRNA (HE667742)	18SMiRT F	–	TCAACGTGCTTGTCCTACCCTGAA	155	60	qRT PCR
		18SMiRT R		TGTGTACAAAGGGCAGGGACGTAA			
17	GFP (HF675000)	gfp F	–	AGCGGCACGACTTCTTCA	750	60	PCR
		gfp R		GTGTGGACAGGTAATGGTTGT			
18	TR10010	FBP_F	fructose bis phosphate aldolase	GCGTCTTCACCTGCATACTT	402	62	PCR
		FBP_R		TAAGGCATTGGCAGACCATC			
19	TR14793	GTfr_F	glucosyl transferase	AGGAATTGCTATTGAGCAGGATA	400	62	PCR
		GTfr_R		GACTGGGACACCAGCATATAAA			
20	TR26363	Msp_F	major sperm protein	CTTCGCGCTCTTCACTCTT	399	62	PCR
		Msp_R		CTTCCGCTTCCGTCCTATTC			
21	TR16177	Asp_F	aspartic protease	CCAGCATCAGATCACGAAGAT	448	62	PCR
		Asp_R		GGTGGAGGATAGGGAGCTATTA			
22	TR10990	Ubq_F	ubiquitin	GTTGTCCTAGAGCCAACACTC	400	62	PCR
		Ubq_R		CGCAATAATGACGATTCGTATGC			

### *Functional validation of role of transcripts in* Meloidogyne – Pasteuria *interaction*

To determine the role of up- and down-regulated transcripts in the *Meloidogyne* ***–*** *Pasteuria* interaction, five out of the 15 transcripts, i.e., TR10010, TR14793, TR26363, TR16177 and TR10990 were selected and silenced by RNAi. Conserved domains in the transcripts were analyzed by NCBI Conserved Domain Database (https://www.ncbi.nlm.nih.gov/Structure/cdd/wrpsb.cgi) and specific primers were designed to amplify them (Table 1). The dsRNA for each of the five transcripts was prepared as described earlier [84]. The effect of dsRNA treatment on the attachment of endospores on nematode cuticle was tested by soaking the J2 s in dsRNA solution at 28 °C for 18 h [105], followed by mixing 100 μl of endospore suspension (2.5 × 10^3^ ml^− 1^) with approximately 200 dsRNA treated J2 s as described earlier [41]. The level of transcript suppression after dsRNA feeding was quantified and analyzed by qRT PCR [84]. Freshly hatched J2 s and J2 s soaked in dsGFP were used as controls. The endospore attachment was quantified by observing ~ 30 nematodes with a Zeiss Axiocam compound microscope (Carl Zeiss, Oberkochen, Germany) and photographed. The assays were performed in triplicate.

## Results

### Transcriptome sequencing and differentially expressed genes

The RNA sequencing of the *Pasteuria* endospore encumbered *M. incognita* J2 s and non-encumbered J2 s generated 32 to 39 million reads per sample (Table 2). The number of high quality reads in the raw sequence data was more than 99% for each of the samples (Table 2). Quality filtering of the raw sequence data using NGS QC resulted in 32.69, 34.62, 39.38 and 37.29 million reads for each of the two samples of non-encumbered and endospore encumbered nematode J2 s, respectively (Table 2). The quality filtered HQ sequence data from all the samples were used to generate a common reference assembly using the Trinity assembler resulting in a total of 161,705 transcripts. The minimum and maximum transcript lengths were 224 and 8320 bp, respectively with the N50 transcript length 1004 bp (Table 3). Improving the assembly by amelioration, removing the duplicates by CD_HIT_EST and filtering un-annotated < 500 bp transcripts resulted in a final merged assembly of 52,485 transcripts (minimum length: 224 bp, maximum length: 8320 bp) with N50 transcript length 1159 bp & GC% of 33.99 (Table 3).

**Table 2:** Raw and Quality filtered data statistics for *Meloidogyne incognita* transcriptomes used in this study. (NE: Non-encumbered nematode J2 s; PE: *Pasteuria* endospore encumbered nematode J2 s; HQ: high quality)

S. No.	Sample name	Total no. of reads	Total no. of bases	Total No. of HQ bases	% HQ Bases	Total HQ reads	Total no of bases in HQ reads	Total no. of HQ bases in HQ reads	% of HQ bases in HQ reads
	RAW					FILTERED			
1	NE-1	32,965,096				32,693,312			
	NE-1.1	16,482,548	1,664,737,348	1,650,917,070	99.17	16,346,656	1,651,012,256	1,639,288,636	99.29
	NE-1.2	16,482,548	1,664,737,348	1,643,633,199	98.73	16,346,656	1,651,012,256	1,635,371,533	99.05
2	NE-2	34,946,244				34,621,924			
	NE-2.1	17,473,122	1,764,785,322	1,752,922,754	99.33	17,310,962	1,748,407,162	1,738,496,781	99.43
	NE-2.2	17,473,122	1,764,785,322	1,741,373,228	98.67	17,310,962	1,748,407,162	1,731,955,805	99.06
3	PE-1	39,837,260				39,382,816			
	PE-1.1	19,918,630	2,011,781,630	1,998,248,213	99.33	19,691,408	1,988,832,208	1,977,968,380	99.45
	PE-1.2	19,918,630	2,011,781,630	1,982,135,511	98.53	19,691,408	1,988,832,208	1,969,001,441	99.00
4	PE-2	37,699,176				37,290,198			
	PE-2.1	18,849,588	1,903,808,388	1,891,336,925	99.34	18,645,099	1,883,154,999	1,872,966,510	99.46
	PE-2.2	18,849,588	1,903,808,388	1,876,575,502	98.57	18,645,099	1,883,154,999	1,864,696,419	99.02

**Table 3:** Assembly statistics of *Meloidogyne incognita* transcriptome generated by Trinity assembler

Parameter	Trinity assembler	Final Ameliorated assembly
Total No. of transcripts assembled	161,705	52,485
Transcriptome length (bp)	113,217,460 (~ 113 Mb)	48,234,212 bp (~ 48.2 Mb)
Min transcript length (bp)	224	224
Max transcript length (bp)	8320	8320
Average transcript length (bp)	700.15	919
N50 contig size (bp)	1004	1159
% (G + C)	33.85	33.99

**Fig. 1:**
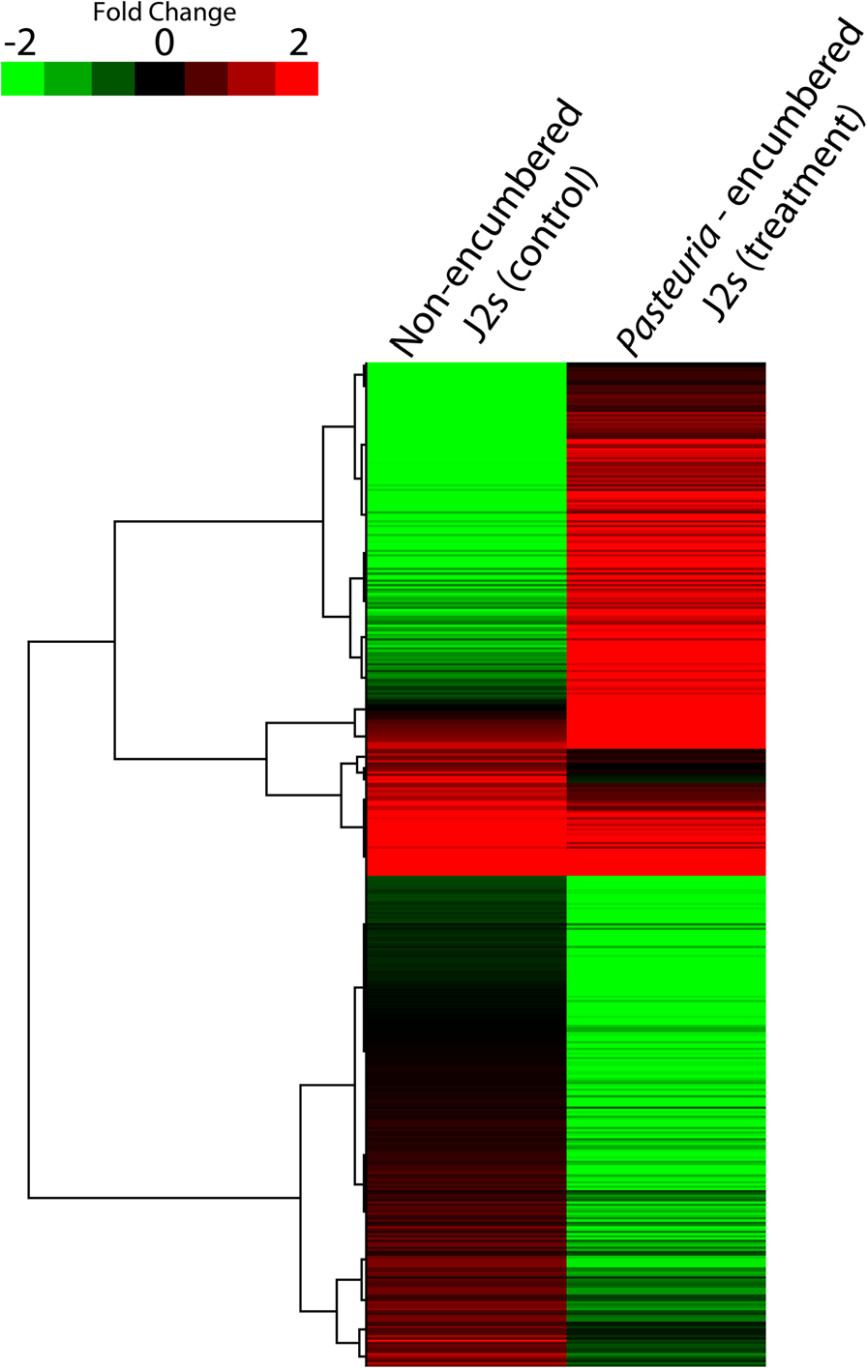
A heat map showing the clustering of the differentially expressed transcripts between non-encumbered and *Pasteuria* encumbered juveniles. The map was generated by using the Log2 fold change values derived from transcriptome data by using DESeq R package between the treatment (endospore encumbered J2 s) and control (Non-encumbered J2 s) groups. The differential expression of transcripts was determined at FPKM > 1(Fragments Per Kilobase of transcript per Million mapped reads) in either of the pair of samples with log2fold change ≥2 with a t-test *P* value ≤0.05

The sequence data from individual samples were analyzed for differentially expressed transcripts between the endospore encumbered and non-encumbered J2 s. Eight hours post attachment of *Pasteuria* endospores, a total of 582 transcripts (1.10% of total transcripts) were found to be differentially expressed, which included 353 down-regulated and 229 up-regulated transcripts (Table 4, Fig. 1, Additional file 1).

**Table 4:** Summary of differentially expressed transcripts in the *Pasteuria* endospore encumbered *Meloidogyne incognita* J2 s (treatment) as compared to the non-encumbered J2 s (control)

S. No.	Parameter	No. of transcripts
1.	Total transcripts	52,485
2.	Annotated	42,511
3.	Differential expression	582
4.	Upregulated	229
5.	Downregulated	353

### Characterization of differentially expressed transcripts

**Fig. 2:**
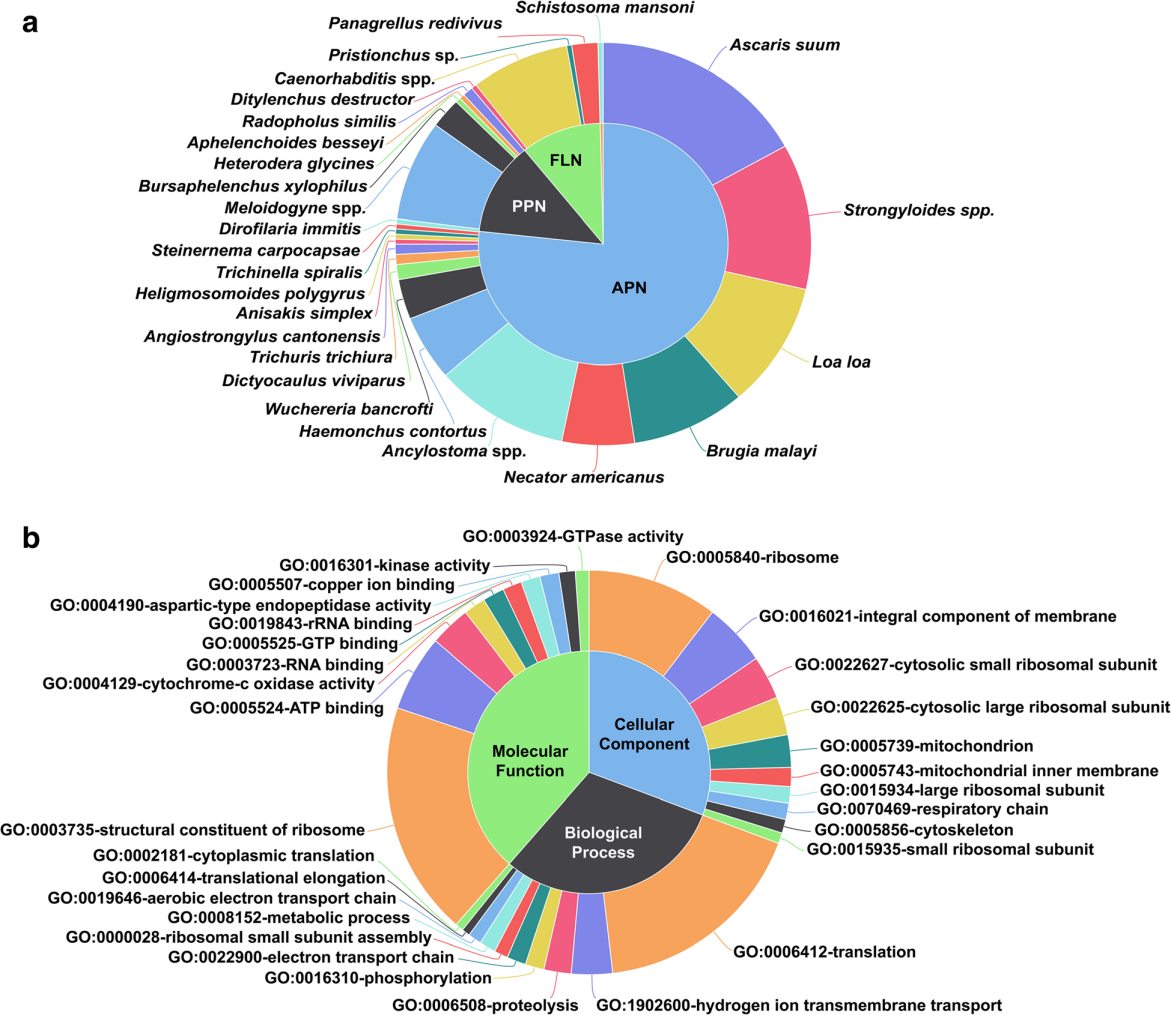
Donut charts showing the annotation of differentially expressed transcripts between the treatment (endospore encumbered J2 s) and control (Non-encumbered J2 s) groups. **a**. Out of 582 differentially expressed transcripts, 246 transcripts showed nematode genes as the top hits. Most of the transcripts matched to those of the animal parasitic nematodes. **b**. The Gene Ontology (GO) of the enriched transcripts. The top ten GO enriched terms under each category of cellular component, biological process and molecular function are represented. APN- animal parasitic nematodes, PPN- plant parasitic nematodes, FLN - free living nematodes

The annotation of 582 differentially expressed transcripts was done by blast search against NCBI nr database. Out of 582 transcripts, 246 transcripts showed nematode genes as the top hits (Fig. 2a). The three most frequent animal-parasitic nematodes found in the blast search were *Ascaris suum* Goeze, 1782 (42 hits), *Strongyloides* spp. (29 hits) and *Ancylostoma* spp. (26 hits), whereas *M. incognita* (12 hits), *Bursaphelenchus xylophilus* (Steiner, 1934) Nickle 1970 (6 hits) and *M. hapla* Chitwood, 1949 (5 hits) were top the top three plant-parasitic nematodes. Nineteen transcripts matched to *Caenorhabditis* spp., whereas one transcript showed the trematode species *Schistosoma mansoni* Sambon, 1907 as its topmost match. The differentially expressed transcripts were further functionally characterized into GO (Gene Ontology) categories of molecular function, biological processes and cellular components. The top ten GO enriched terms under each category are represented in Fig. 2b.

The transcripts showing highest down-regulation in the RNA-Seq experiment, TR38275 (− 7.0 folds) and TR22780 (− 6.9 folds) were annotated as heat shock protein 90 (HSP 90) and papain family cysteine protease, respectively (Additional file 1). The biggest group of down-regulated proteins were different ribosomal proteins (79 transcripts), whereas, hypotheticals represented the second largest group of the down-regulated proteins (49 transcripts). The heat shock proteins represented the third biggest group of down-regulated proteins (23 down-regulated transcripts) and included proteins such as HSP 70, HSP 20 and HSP 12.6. Eleven protease/proteinase (including two cysteine proteinases) and six kinases/phosphatases also showed down-regulation. Some other interesting proteins showing down-regulation were plectin (2 transcripts) and secretory proteins (3 transcripts), two fructose bisphosphate aldolase class-I family, one fatty acid and retinol binding protein, one signal transduction protein possibly involved in cell surface receptor signaling pathway (GO:0007166), three ubiquitin related proteins, three homologues of phospholipase B like 2 and one of phospholipase A2. In addition, *M. incognita* genes showing homology to tropomyosin (2 transcripts), actin-2 and tubulin (1 transcript each) were also found to be substantially down-regulated (Additional file 1).

The top three highly up-regulated transcripts in *Pasteuria* encumbered J2 s were TR25864, TR1903 and TR25239 that showed 9.4, 9.3 and 8.9 fold up-regulation in the RNA-Seq experiment. All of these three transcripts were uncharacterized or hypothetical proteins. In fact, hypotheticals, uncharacterized and unknown proteins were the dominant group under the up-regulated transcripts with 96 out of total 229 up-regulated transcripts falling into these categories. Cytochrome oxidase and major sperm protein domain containing proteins were the second most enriched transcript groups with 13 transcripts each. Blast based annotation showed that five transcripts matched to hormone receptor-like in 38 genes, four to transformation transcription domain-associated protein, two to venom allergen-like protein and one transcript matched to TK/FER protein kinase, UDP-glucosyl transferase and glutathione-S-transferase each (Additional file 1).

Analysis of the pathways represented by transcripts differentially expressed in the encumbered J2 s was performed by KAAS, an automatic genome annotation and pathway reconstruction tool. Annotation of the differentially expressed transcripts by KAAS showed that out of 582 transcripts, 148 could be mapped to 153 different pathways (Additional file 2). Ribosome (KO03010) was the most highly active pathway with 62 active transcripts, followed by thermogenesis (KO04714, 8 transcripts). A list of top 20 pathways and number of transcripts mapped to those pathways are shown in Table 5. A number of signaling pathways were active that are known to be involved in development and diverse functions in the encumbered J2 s such as ErbB signaling pathway (KO04012), Wnt signaling pathway (KO04310), Hedgehog signaling pathway (KO04341), TGF-beta signaling pathway (KO04350), Hippo signaling pathway (KO04390, KO04391), Apelin signaling pathway (KO04371), FoxO signaling pathway (KO04068), Rap1 signaling pathway (KO04015), PI3K-Akt signaling pathway (KO04151), AMPK signaling pathway (KO04152), cAMP signaling pathway (KO04024), cGMP-PKG signaling pathway (KO04022), MAPK signaling pathway (KO04010), mTOR signaling pathway (KO04150), HIF-1 signaling pathway (KO04066), calcium signaling pathway (KO04020), and sphingolipid signaling pathway (KO04071). In addition, pathways involved in recycling of cellular contents such as endocytosis (KO04144), phagosome (KO04145), lysosome (KO04142) and autophagy-animal (KO04140) were also found to be active in the encumbered J2 s.

**Table 5:** The top 20 pathways active in the endospore encumbered *Meloidogyne incognita* J2 s at 8 h post exposure, and the numbers of transcripts mapped to these pathways

S. No.	KEGG Pathway ID (KO)	Pathway	No. of mapped transcripts
1.	03010	Ribosome	62
2.	04714	Thermogenesis	8
3.	05012	Parkinson’s disease	7
4.	00190	Oxidative phosphorylation	6
5.	04210	Apoptosis	6
6.	05016	Huntington’s disease	6
7.	00620	Pyruvate metabolism	5
8.	04142	Lysosome	5
9.	04910	Insulin signaling pathway	5
10.	04260	Cardiac muscle contraction	5
11.	05205	Proteoglycans in cancer	5
12.	05010	Alzheimer’s disease	5
13.	00010	Glycolysis / Gluconeogenesis	4
14.	04141	Protein processing in endoplasmic reticulum	4
15.	04151	PI3K-Akt signaling pathway	4
16.	04152	AMPK signaling pathway	4
17.	04217	Necroptosis	4
18.	04932	Non-alcoholic fatty liver disease (NAFLD)	4
19.	04144	Endocytosis	3
20.	04145	Phagosome	3

The search for differentially regulated secreted peptides revealed that a total of 376 differentially expressed transcripts contained secretion signal, out of which 90 transcripts showed up-regulation, whereas 286 showed down-regulation. Of these 376 transcripts, only 320 could be annotated (Table 6, Additional file 2).

**Table 6:** Number of transcripts with secretion signal. Out of 376 differentially expressed transcripts, 320 showed presence of a secretion signal

Sl.	GO categories	No. of transcripts
1.	Without GO term	101
2.	Ribosomal structure	79
3.	Peptidase	22
4.	Transferase	21
5.	Protein binding	18
6.	Glycosyl transferase	16
7.	DNA binding	14
8.	Metal ion binding	10
9.	GTPase activity	9
10.	Oxidoreductase	7
11.	Hydrolase	3
12.	Catalytic	3
13.	Ion transporter	3
14.	Heme binding	2
15.	Calcium binding	2
16.	Exonuclease	2
17.	Lipid binding	1
18.	Protein kinase	1
19.	Sodium channel activity	1
20.	Galactosyl transferase	1
21.	Fucosyl transferase	1
22.	Asparagine synthase activity	1
23.	GPCR activity	1
24.	Protein phosphate regulator activity	1

### Validation of differentially expressed transcripts by qRT PCR

A total of fifteen transcripts, identified as differentially expressed in the RNA-Seq data, were taken up for validation by qRT PCR. Expression of 12 out of 15 transcripts was validated as significantly up- or down- regulated at 8 h post endospore encumbrance (Table 7). Unlike the RNA-Seq data, the fold change expression of the transcripts encoding heat shock protein 20, glutathione S-transferase and tropomyosin was not found to be significant. Data revealed that the selenium binding protein coding transcript (TR31579) showed maximum up-regulation by 6.89 fold, followed by the transcripts coding for glucosyl transferase (TR14793; 4.89 fold) and major sperm protein (TR26363; 2.92 fold), as compared to control. The transcripts showing highest down-regulation were TR14120 (phospholipase A2; − 7.98 fold), TR10010 (fructose bisphosphate aldolase; − 7.63fold) and TR11544 (fatty acid and retinol binding protein; − 6.09 fold), as compared to control. A comparison of the fold changes as detected by RNA-Seq and qRT PCR is provided in Table 7.

**Table 7:** Comparison of fold expression of selected transcripts between RNA-Seq and qRT PCR experiments. The *P* values for the RNA-Seq data is provided in parentheses, and the statistical significance of the qRT PCR data is indicated by superscripted letters (a,b)

S. No.	Transcript ID	Annotation	Fold Change	
			RNA-Seq	qRT PCR
1.	TR11426	Heat shock protein 20	−4.18 (*P* = 0.02)	−1.24
2.	TR14120	Phospholipase A2	−2.78 (*P* = 0.01)	−7.98^b^
3.	TR16177	Aspartic protease	−3.93 (*P* = 0.00)	−2.52^b^
4.	TR10194	Bm 3887	−2.10 (*P* = 0.05)	−3.20^b^
5.	TR10010	Fructose bis phosphate aldolase	−3.60 (P = 0.00)	−7.63^b^
6.	TR31579	Selenium binding protein	2.01 (P = 0.00)	6.89^b^
7.	TR14793	Glucosyl transferase	2.34 (*P* = 0.04)	4.89^b^
8.	TR23171	Venom allergen-like protein	2.68 (P = 0.02)	1.73^a^
9.	TR35213	Glycoside hydrolase	−3.89 (P = 0.00)	−4.16^b^
10.	TR40461	Glutathione S-transferase	2.13 (P = 0.00)	0.51
11.	TR11544	Fatty acid and retinol binding protein	−5.06 (P = 0.00)	−6.09^b^
12.	TR26363	Major sperm protein	3.46 (P = 0.00)	2.92^b^
13.	TR10990	Ubiquitin	− 4.82 (P = 0.00)	−5.04^b^
14.	TR20164	Tropomyosin	−3.32 (P = 0.00)	−0.35
15.	TR24005	Serine protease	−3.96 (*P* = 0.00)	−2.71^b^

^a^significant and ^b^highly significant as compared to control. Data was considered statistically significant at *P* value ≤0.05 for both the experiments

### *Functional evaluation of role of M. incognita genes on* Pasteuria *encumbrance by RNAi*

**Fig. 3:**
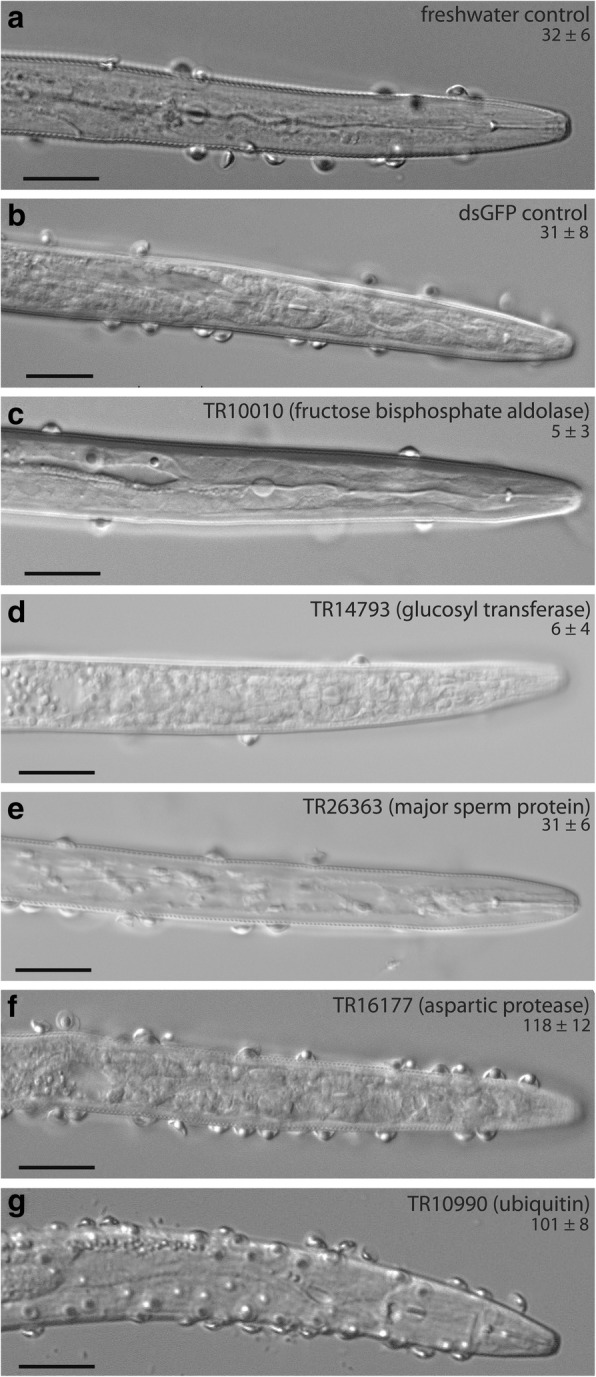
Effect of RNAi induced gene silencing of five transcripts on the attachment of *Pasteuria penetrans* endospores (seen as hemispherical structures) sticking onto *Meloidogyne incognita* J2 s. (**a**). non-silenced freshwater control J2 s, (**b**). dsGFP treated control J2 s, (**c** to **g**) – J2 s in which specific transcripts were RNAi silenced. The transcript ID and the average number of attached endospores are provided along with the name of the silenced genes. Silencing of TR10010 and TR14793 caused significantly reduced endospore attachment as compared to controls, whereas silencing of TR16117 and TR10990 caused a significant increase in the endospore attachment. Silencing of TR26363 did not cause any change in endospore attachment as compared to controls. (scale bar: 20 μm)

Five transcripts were selected for functional validation based on their fold expression and predicted role in *Meloidogyne – Pasteuria* interaction. These included three down-regulated transcripts, viz., TR10010 (fructose bisphosphate aldolase), TR16177 (aspartic protease) and TR10990 (ubiquitin); and two up-regulated transcripts TR14793 (glucosyl transferase) and TR26363 (major sperm protein). The qRT PCR result showed that dsRNA treatment caused significant down-regulation of TR10010, TR16177, TR10990, TR14793 and TR26363 expression by 3.97 ± 0.26, 4.21 ± 0.37, 4.68 ± 0.12, 3.45 ± 0.33 and 2.95 ± 0.40 folds, respectively, as compared to the control. The dsRNA induced silencing of fructose bisphosphate aldolase (5 ± 3 endospores/J2) and glucosyl transferase (6 ± 4 endospores/J2) resulted in approximately six times lower endospore attachment as compared to the controls (water control: 32 ± 6 and dsGFP control: 31 ± 8 endospores/J2). On the other hand, silencing of aspartic protease (118 ± 12 endospores/J2) and ubiquitin (101 ± 8 endospores/J2) resulted in approximately three fold higher incidence of endospore attachment. However, there was no change on endospore attachment in the major sperm protein silenced J2 s (31 ± 6 endospores/J2), as compared to the controls (Fig. 3).

## Discussion

Present RNA-Seq experiment provides a snapshot of the early molecular response by the *M. incognita* J2 s after exposure to *P. penetrans* endospores. Our results show that 582 nematode transcripts were differentially expressed at 8 h post *Pasteuria* endospore attachment of which 229 were up-regulated, and 353 were down-regulated. The expression patterns of 12 out of 15 differentially expressed (ten down- and five up-regulated) transcripts identified by the RNA-Seq were significantly validated to be true by qRT PCR. The functional role of five transcripts predicted to be important in *Meloidogyne – Pasteuria* interaction were validated by RNAi. The silencing of transcripts coding for fructose bisphosphate aldolase (TR10010) and glucosyl transferase (TR14793) resulted in approximately six times lower endospore attachment as compared to the controls, whereas, silencing of aspartic protease (TR16177) and ubiquitin (TR10990) coding transcripts resulted in approximately three fold higher incidence of endospore attachment. No change on endospore attachment was detected in major sperm protein (TR26363) silenced J2 s.

As established in several natural host-parasite interactions, viz., *Daphnia – Pasteuria*, *Anopheles – Plasmodium* and Bumblebee *– Trypanosoma*, a successful infection results from a compatible molecular interaction between the host and parasite genotypes [6, 55, 56, 64]. *Daphnia magna* Straus, 1820 responds to *P. ramosa* Metchnikoff, 1888 infection by rapid gene expression at 4 h, and the response tapers off with the passage of time at 8 and 12 h [64]. However, unlike *Daphnia*, which becomes infected after *Pasteuria* is orally ingested and adheres to the esophageal region, *Meloidogyne* becomes infected when *Pasteuria* spores adhere to the juvenile body surface and germinate when the nematode starts to feed on plants. In a study on *Meloidogyne – Pasteuria* interaction at three days post endospore adherence, Zou et al. [117] found that nematodes respond by an up-regulated gene expression, and identified 445 differentially expressed genes out of which 406 were up-regulated while 39 got down-regulated. Thirty seven immune responsive genes encoding collagens, cytochrome P450, lysozymes were among the other identified active proteins. Interestingly, Zou et al. [117] found that biosynthesis of unsaturated fatty acid pathways was up-regulated, while cytochrome P450 related genes were down-regulated. However, the findings from the *Daphnia – P. ramosa* system suggest that there may be an even earlier transcriptional response; hence, we focused on understanding the early transcriptional response by the nematode at eight hours after endospore attachment.

Our study showed that 153 pathways were active in *Pasteuria* encumbered *M. incognita* J2 s at 8 h post infection. As compared to our finding, 98 pathways were enriched at 3 days post *Pasteuria* attachment [117], of which 58 pathways were found to be active at both the time points (Additional file 2). ErbB signaling, Rap1 signaling, FoxO signaling, Hedgehog signaling, thermogenesis, longevity regulating pathways were some of the pathways active only at 8 h post endospore attachment; whereas pathways like cytochrome P450 (drug and xenobiotic metabolism), MAPK signaling, calcium signaling, HIF-1 signaling, mTOR signaling, PI3K-Akt signaling, Wnt signaling, TGF-beta signaling, Hippo signaling and regulation of actin cytoskeleton were active at both the time points. Some of the pathways active at 8 h assist cell-cell interaction and cellular adhesion in model organisms including *C. elegans*, for example, ErbB signaling [113], TGF-beta signaling [39], Hippo kinase cascade [114] and Rap1 signaling [8]. Other pathways like Wnt signaling [97], FoxO signaling [40], PI3K-Akt signaling [33], AMPK signaling [68], MAPK signaling [47], mTOR signaling [43] and HIF-1 signaling [116] operate in bacterial pathogenesis and stress responses. Taken together with the findings of Zou et al. [117], it appears that *M. incognita* immune pathways like cytochrome P450, MAPK signaling, HIF-1 signaling, mTOR signaling, PI3K-Akt signaling and TGF-beta signaling pathways were active upon infection by *Pasteuria* from 8 h to 3 days, in addition to the regulation of actin cytoskeleton pathway (Additional file 2).

Our study indicates that the nematode’s transcriptional responses after *Pasteuria* infection can be broadly understood as: (a) transcripts involved in nematode immunity, (b) transcripts involved in altering the cuticular surface coat property and thereby affecting endospore attachment, (c) transcripts involved in modulating the behaviour of the endospore encumbered juveniles.

### *Nematode immune responses triggered by* Pasteuria

In addition to the immune pathways discussed above, at 8 h post endospore encumbrance, the ribosomal pathway (KO03010) was identified as the most affected with 62 differentially expressed transcripts. Besides, the ribosomal proteins represented the largest down-regulated group (79 transcripts) along with three ubiquitin related transcripts and one ZIP or ZRT/IRT-like protein (TR38155). The suppression of these transcripts suggest a reduction in the mRNA translational activity in *Pasteuria* encumbered *M. incognita* J2 s. This is consistent with the earlier reports of “effector-triggered” or “surveillance immunity”, where a down-regulation of host mRNA translation upon attack by bacterial pathogens has been reported in plants and in *C. elegans* [20, 29, 63, 67]. The bacterial pathogens disable the process of host mRNA translation, thereby preventing the production of anti-microbial molecules, and improve the chances of the infection [20]. It appears that a similar strategy is being used by *Pasteuria* while infecting *M. incognita.* The RNAi mediated knockdown of nematode ubiquitin also increased the endospore attachment on cuticular surface. Several bacterial pathogen effectors interact with eukaryotic ubiquitination pathways to exploit host functions [80]. The involvement of ubiquitin proteasome system, targeting proteins for degradation, has been established in *C. elegans* as an inducible response to infection [4, 20, 65]. Silencing of ubiquitin may lead to anomalous immune response where the cells fail to mount a sufficient immune response to remove the pathogen.

Approximately 13 autophagy related transcripts (e.g. endocytosis, KO04144; phagosome, KO04145; lysosome, KO04142 and autophagy-animal, KO04140) were down-regulated in the endospore encumbered *M. incognita* J2 s. It is well established that autophagy plays key role in pathogen defense [20]. Further, a down-regulation of 23 heat shock protein coding transcripts, for example, HSP70, HSP20 and HSP12.6 was observed. The HSPs are highly conserved group of proteins and are involved in protection against biotic and abiotic stress [19, 32, 44, 81, 91, 95, 115]. Similarly, HSP70s and HSP20 are known to play crucial role during disease stress response and serves as an endogenous danger signal [10, 23, 98]. Although down-regulation of HSPs during biotic stress is rare [78]; small HSPs (e.g. HSP17, HSP21 etc.) and HSP70 were found to be down-regulated in *Arabidopsis* when challenged by *Pseudomonas syringae* van Hall, resulting in suppression of host defense responses [10]. The down-regulation of autophagy related genes and HSPs by *P. penetrans* indicates pathogen induced host defense suppression.

In addition to the above mentioned pathways, several differentially expressed transcripts related to nematode immunity against bacterial pathogens were identified, such as, aspartic protease (TR16177), phospholipase A2 (TR14120), glutathione S-transferase (TR40461), selenium binding proteins (TR31579) and hormone receptor-like in 38 (Hr38) (TR5128, TR39260). Aspartic proteases function in the intracellular and extracellular degradation of proteins including processing of peptide hormones, antigens and immunoglobulins in parasitic nematodes [51, 100, 101]. In the present study, the RNAi induced inhibition of aspartic protease led to an increase in *Pasteuria* endospore adhesion on nematode cuticle indicating the defensive role of aspartic protease against bacterial pathogens. The inhibition of nematode phospholipase A2 by *Pasteuria* possibly prevents the bacterial infection structures from degradation, thereby allowing further infection [89]. Glutathione S-transferase (GST) was up-regulated in *Pasteuria* infected J2 s. In *C. elegans* and parasitic helminthes, GST is associated with an immune response and xenobiotic metabolism [11, 12, 18, 46, 62, 107, 108]. The up-regulation of GST may indicate nematode’s efforts to detoxify the potential *Pasteuria* effectors [86]. The RNAi mediated knockdown of *M. incognita* selenium binding protein (SeBP) significantly increased *P. penetrans* endospore attachment possibly through altering the cuticular surface coat property [83]. Lastly, the nuclear receptors (NRs) are ligand-dependent transcription factors that play pivotal roles in cell growth, differentiation, metabolism, reproduction and morphogenesis and immunity [13, 34]. Up-regulation of hormone receptor-like in 38 (Hr38) transcript, the *Drosophila* ortholog of the mammalian NGFI-B subfamily of orphan nuclear receptors [16] in *M. incognita* may be an indication of detection of the foreign nucleic acid particle (of *Pasteuria*), thereby triggering an immune response in *M. incognita*.

### *Modification of nematode cuticle biochemistry by* Pasteuria

Mutations in genes involved in the building of complex cuticular surface components in *C. elegans* are known to affect bacterial adhesion [37]. Several differentially expressed transcripts identified in this study are predicted to interfere with attachment of endospores on the nematodes by altering the cuticle surface structure. These include TR10010 (fructose bisphosphate aldolase), TR14793 (glucosyl transferase), TR11544 (fatty acid and retinol binding (FAR) protein), and TR24724 (TK/FER kinase).

The fructose bisphosphate aldolase is a key enzyme of the glycolytic pathway [79] and its inhibition may contribute to accumulation of sugar molecules on the cuticle surface [30]. Similarly, glucosyl transferase catalyzes the transfer of sugar moieties to a wide range of acceptor molecules [73, 112]. RNAi induced in vitro inhibition of both these transcripts reduced the endospore attachment on nematode surface. Silencing fructose bisphosphate aldolase might have resulted in accumulation of sugar on the specific carbohydrate recognition sites on nematode mucin-like glycoprotein that binds to *Pasteuria* endospores [25, 85], thereby leading to reduction in attachment of endospores. Additionally, inhibition of glucosyl transferase may also result in mis-folding of the native glycoprotein molecules, thus resulting in decreased endospore attachment. Our findings are in concurrence with earlier observations on *C. elegans*, where the *bus-8* mutant worms, defective in expressing glucosyl transferase were resistant to infection by *Microbacterium nematophilum* Hodgkin, Kuwabara and Corneliussen, 2000, due to failure of the bacterium to bind to the host surface [76]. The fatty acid and retinol binding (FAR) protein, uniquely present in nematodes, has been found to inhibit bacterial attachment onto nematode body surface by sequestering pharmacologically active lipids [45]. It has been demonstrated that the FAR protein is involved in the protection of *M. incognita* from attachment of *P. penetrans* endospores [84]. The TK/FER kinase regulates cadherin and integrin dependent cellular adhesion [9] and is also involved in nascent cell-cell adhesion via phosphorylation pathway [50]; and our results suggest it may also be involved in *M. incognita* and *P. penetrans* infection processes.

### *Alteration of nematode behavior and locomotion by* Pasteuria

The endospore encumbered stressed nematode juveniles are slower in movement and finding their hosts [25, 84, 106]. The role of tropomyosin, actin and tubulin in regulation of muscle contraction has been established for several plant-parasitic, animal-parasitic and free living nematodes [38, 54]. As observed in our RNA-Seq data, down-regulation of these muscle-associated proteins upon endospore attachment may result in perturbed locomotion of the nematodes. The RNA-Seq data showed perturbation in a large number of neuropeptides in the *Pasteuria* encumbered juveniles in our study (Additional file 2). The neuropeptides of major groups, viz., FLPs, NLPs and ILPs affect numerous behavioral responses via various signaling pathways [14, 17, 21, 52, 109]. The association of FLPs and NLPs with nematode locomotion has been observed in *C. elegans* [48, 70, 90] and ILPs in the fruit fly movement [31]. Disruption of *flp-18* in *M. incognita* by RNAi is known to reduce the plant parasitism [75]. The nematode behavioral alteration could also result from the effect of *Pasteuria* on the neuropeptides, as suggested by our results.

Apart from the above mentioned pathways and transcripts, two interesting observations from our study need mention. Firstly, we found that a major sperm protein (MSP) was up-regulated in infected juveniles, but RNAi mediated silencing of MSP coding transcript (TR26363) did not affect the adhesion of *Pasteuria* endospores onto nematode cuticle surface. MSP is involved in motility machinery and crawling movement of nematode sperms [92, 94], and is regulated at the onset of sexual differentiation in nematodes [49]. In *C. elegans*, MSP promotes the oocyte maturation and MAPK activation [53, 66]. It is well known that *Pasteuria* is largely confined to the reproductive system of *M. incognita* leading to complete destruction of its reproductive ability [7]. The sexual differentiation and gonad development process in *M. incognita* starts in the second-stage juveniles (J2 s) [74] and the up-regulation of MSP by *Pasteuria* might indicate an early interference with the nematode’s reproductive system.

Secondly, it has been observed that *Pasteuria* infected *M. incognita* have an increased life span of 10–12 days [25, 82], which could be a consequence of reduced mRNA translation in microbial infection. This corroborates with other studies in which the down-regulation of protein synthesis has resulted in increased lifespan [35], possibly by two different mechanisms. Firstly, reduced mRNA translation decreases the synthesis of normal as well as damaged proteins, resulting in lower accumulation of toxic proteins [42]. Secondly, as protein synthesis is a high cellular energy-consuming process [57], reduction of mRNA translation might increase energy availability and allow diversion of critical resources towards cellular maintenance and repair, thus promoting longevity [102, 103]. Our study shows a major reduction in protein synthesis in the *Pasteuria* encumbered nematodes thereby possibly extending lifespan when compared to the healthy nematodes.

Lastly, the hypothetical proteins represented the second largest group of the down-regulated proteins with 49 transcripts. There could be several interesting candidates within this group that may be directly or indirectly involved in the RKN – *Pasteuria* interaction.

## Conclusion

Here we presented a transcriptomic analysis of *M. incognita* and *P. penetrans* interaction at eight hour post initial encounter. The transcriptome profile revealed that *Pasteuria* infection causes a reduction of the protein synthesis machinery of the nematode. We identified several differentially expressed transcripts putatively involved in nematode innate immunity, endospore attachment process and post-attachment behavioral modification of the juveniles. RNAi based functional validation of fructose bisphosphate aldolase (TR10010), glucosyl transferase (TR14793), aspartic protease (TR16177) and ubiquitin (TR10990) coding transcripts resulted in altered incidence of endospore attachment, whereas silencing of major sperm protein (TR26363) did not result in any alteration in *Pasteuria* attachment. Our results add new and significant information to the existing knowledge on early molecular interaction of *M. incognita* and *P. penetrans*.

## Additional files

Additional file 1: A list of all, and the differentially expressed transcripts between endospore encumbered and non-encumbered *Meloidogyne incognita* juveniles at 8 hours post-*Pasteuria* attachment. (XLSX 7442 kb)

Additional file 2: KAAS pathway mapping, comparison of pathways active at 8 hours and 3 days, and annotation of neuropeptides and secreted peptides in the differentially expressed transcripts. (XLSX 534 kb)

## Abbreviations

GO: gene ontology
J2: second stage juvenile
KAAS: KEGG Automated Annotation Server
KEGG: Kyoto Encyclopedia of Genes and Genomes
qRT PCR: quantitative real time PCR
RKN: root-knot nematodes
RNAi: RNA interference
